# Association of Dietary Advanced Glycation End Products with Overall and Site-Specific Cancer Risk and Mortality: A Systematic Review and Meta-Analysis

**DOI:** 10.3390/nu17101638

**Published:** 2025-05-10

**Authors:** Carlos Pascual-Morena, Miriam Garrido-Miguel, Irene Martínez-García, Maribel Lucerón-Lucas-Torres, Eva Rodríguez-Gutiérrez, Carlos Berlanga-Macías, Jaime Fernández-Bravo-Rodrigo, Silvana Patiño-Cardona

**Affiliations:** 1 Health and Social Research Center, Universidad de Castilla-La Mancha, 16071 Cuenca, Spain; carlos.pascual@uclm.es (C.P.-M.); miriam.garrido@uclm.es (M.G.-M.); mariaisabel.luceron@uclm.es (M.L.-L.-T.); eva.rodriguez@uclm.es (E.R.-G.); carlos.berlanga@uclm.es (C.B.-M.); silvana.patino@alu.uclm.es (S.P.-C.); 2 Facultad de Enfermería de Albacete, Universidad de Castilla-La Mancha, 02006 Albacete, Spain; 3 CarVasCare Research Group, Facultad Enfermería de Cuenca, Universidad de Castilla-La Mancha, 16071 Cuenca, Spain; jaime.fernandezbravo@alu.uclm.es; 4 Pharmacy Service, Hospital Virgen del Castillo, 30510 Yecla, Spain

**Keywords:** dietary advanced glycation end products, pro-inflammatory diet, oxidative stress, site-specific cancer, systematic review, meta-analysis

## Abstract

**Background/Objectives**: Dietary advanced glycation end products (dAGEs) have a pro-inflammatory effect and increase oxidative stress, potentially leading to cancer. The aim of this study was to estimate the association between dAGEs consumption and risk and mortality from overall cancer and according to its site. **Methods**: A systematic search was conducted in Medline, Scopus, Web of Science, and the Cochrane Library from inception to April 2025. The search strategy was conducted according to the PECO structure adapted to this study, as well as the inclusion criteria, in which the population (P) was the adult population, the exposure (E) was the highest level of dAGEs intake, the comparator (C) was the lowest level of dAGEs intake, and the outcomes (O) were the overall cancer risk, cancer risk by site, and cancer mortality. Results across studies were summarised using random effects and fixed effects. **Results**: Fourteen studies were included in the systematic review. In the random-effects meta-analysis, high dAGEs intake was associated with Hazard Ratio (HR) = 0.99 [95% Confidence Interval (95% CI): 0.98, 1.00] for overall cancer risk. However, although there was no association with breast cancer (BC), there was an association with invasive BC, with HR = 1.14 (95% CI: 1.05, 1.23). In contrast, in other tumours, there were opposite results depending on the site of the cancer. **Conclusions**: The reduction in cancer risk is not clinically significant. However, high consumption of dAGEs may increase the risk of BC, particularly the invasive BC, which is a challenge for cancer prevention and subsequent mortality. Due to the limited evidence, further studies are needed to confirm the potential impact of dAGEs, as well as other dietary factors that may play a larger role in cancer development.

## 1. Introduction

Cancer is a leading cause of morbidity and mortality worldwide, with an estimated 19.3 million new cases and 10 million deaths worldwide in 2020 [1]. The increase in incidence is due to both non-modifiable factors, such as age, sex, and genetic susceptibility, and modifiable factors, which are related to lifestyle [2]. Unlike non-modifiable factors, modifiable factors can be controlled to reduce the risk of cancer. These include harmful habits such as smoking, sedentary lifestyles with little or no physical activity, and the consumption of processed foods with high energy density and low nutritional value, which contribute to the development of diseases such as obesity, type 2 diabetes mellitus, heart disease and cancer, among other chronic diseases [3,4,5,6]. Breast cancer (BC) is the leading cause of cancer incidence worldwide, accounting for nearly 30% of all cancers diagnosed in women in the European Union. Although genetic predisposition plays an important role, up to a quarter of BC cases can be prevented by improving modifiable factors [2,3,5].

One group of compounds that may be carcinogenic are advanced glycation end products (AGEs). AGEs are reactive metabolites formed endogenously as a result of several metabolic pathways, although they are also ingested with food. These compounds are abundant in highly processed, sugary, fatty, or high-temperature cooked foods and are formed by irreversible non-enzymatic reactions between sugars, proteins, and lipids [7,8,9]. Evidence suggests that AGEs may play a role in cancer development through their pro-inflammatory and pro-oxidant properties by the stimulation of the receptor for advanced glycation end products (RAGEs). Thus, AGEs bind to RAGEs and activate pro-inflammatory pathways such as NF-κB and MAPK, which in turn increase the transcription of IL-6, TNF-α, and CCL2, creating a chronic inflammatory microenvironment that favours tumour cell proliferation. In addition, this process generates reactive oxygen species (ROS) through activation of NADPH oxidase and mitochondrial dysfunction, causing DNA damage, lipid and protein oxidation, and promoting mutagenesis [10,11,12].

In the foods, there are several types of dietary AGEs (dAGEs), the most important of which are N-ε-[carboxymethyl]-L-lysine (CML), formed by glycation of lysine in proteins and aminolipids and by lipid peroxidation reactions, N-ε-(carboxyethyl)-L-lysine (CEL), formed via reaction of lysine with methylglyoxal, pentosidine, a cross-linking AGE from pentose sugars and arginine/lysine, and N-δ-[5-hydro-5-methyl-4-imidazolon-2-yl]-ornithine (MG-H1). These are mainly found in foods such as meat and proteins of animal origin, dairy products, sausages, fried foods, and pastries. These dietary AGEs, in addition to their pro-oxidative potential via the pathways described above, can promote insulin resistance and increase tissue stiffness by forming cross-links with collagen and other matrix components, potentially promoting the development of various diseases, including cancer [9,13].

A previous review revealed no association between dAGEs intake and overall cancer risk [14]. In their sensitivity analyses, they also found no association for BC, pancreatic cancer, colon cancer, and rectal cancer. However, they did not examine the impact of cancer mortality, nor did they examine it by BC subtypes, whether invasive or not, or by hormone receptor (oestrogen and progestogen). Finally, the possibility of including other studies could not be ruled out. Therefore, the aim of this systematic review and meta-analysis was to estimate and assess the association between dAGEs and overall risk and/or mortality from cancer and according to its site.

## 2. Materials and Methods

This study was conducted following the guidelines for Preferred Reporting Items for Systematic Reviews and Meta-Analyses (PRISMA), Meta-analysis of Observational Studies in Epidemiology (MOOSE), and the Cochrane Collaboration Handbook [15,16,17]. The study was registered in PROSPERO (registration ID: CRD42024627587).

### 2.1. Search Strategy

A systematic search was conducted in Medline (via PubMed), Scopus, Web of Science, and the Cochrane Library, as well as an open search of the grey literature, including the OpenGrey, Google Scholar, ProQuest, and Networked Digital Library of Theses and Dissertations databases, from inception to April 2025. References from previous reviews and included studies were also screened. Finally, the authors of the studies were contacted if the full text was not available. The search strategy is detailed in Appendix S1.

Systematic searches were carried out independently by two authors (CP-M and SP-C), and disagreements were solved by consensus or by a third author (IM-G).

### 2.2. Inclusion/Exclusion Criteria

Observational studies estimating the association between dietary AGEs intake and cancer risk and mortality from cancer were included.

Inclusion criteria were as follows: (1) population: adults; (2) exposure: the highest tertile, quartile, or quintile of dAGEs intake; (3) comparator: the lowest tertile, quartile, or quintile of dAGEs intake; (4) outcomes: overall cancer risk, cancer risk by location, and mortality from cancer; (5) design: observational studies, including prospective, retrospective, and case-control studies.

Exclusion criteria were as follows: (1) outcomes: studies estimating the risk of cancer relapse or recurrence; (2) design: case series studies. There were no language restrictions.

Study selection was performed independently by two authors (CP-M and SP-C), and disagreements were solved by consensus or by a third author (IM-G).

### 2.3. Data Extraction

An ad hoc table was created with the following data extracted from the included studies: (1) reference (author and year); (2) region (country/ies or continent); (3) design, including cohorts (i.e., prospective or retrospective) or case-control; (4) sample size and percentage of females; (5) age (mean or median); (6) comparisons (tertiles, quartiles, quintiles); (7) type of dAGE measured; (8) follow-up; (9) outcomes (overall cancer risk, risk of cancer by location, mortality from cancer, other cancer mortality).

Data extraction was performed independently by two authors (CP-M and SP-C), and disagreements were solved by consensus or by a third author (IM-G).

### 2.4. Risk of Bias Assessment

The Study Quality Assessment Tools from the United States National Institute of Health National Heart, Lung, and Blood Institute were used to assess the risk of bias of the included studies, for cohort and case-control studies separately [18,19]. For cohort studies, the tool has 14 methodological and statistical items, whereas the tool for case-control studies has 12 items. In both cases, the overall risk of bias was good if <2 items were rated as poor, fair if 2 items were rated as poor, and poor if >2 items were rated as poor.

Risk of bias was assessed independently by two authors (CP-M and SP-C), and disagreements were solved by consensus or by a third author (IM-G).

### 2.5. Grading the Quality of Evidence

The quality of the evidence was assessed using the Grading of Recommendations, Assessment, Development, and Evaluation (GRADE) tool [20]. This tool scores the strength of the evidence for each outcome from high to very low based on several domains. The GRADE tool assesses the quality of scientific evidence by considering several key areas: the number and design of included studies (with greater emphasis on randomised controlled trials than observational studies); the risk of bias in the studies; inconsistency of results (e.g., high heterogeneity between studies); indirect evidence (when studies do not directly address the population, intervention or outcome of interest); imprecision of results (e.g., estimates with wide confidence intervals); and other factors such as the size of the effect observed or the possible presence of publication bias.

### 2.6. Statistical Analysis

An ad hoc table and a narrative synthesis of the results of each study were performed. The results were expressed as a hazard ratio with their 95% confidence intervals (95% CI). The 95% CI was extracted directly from the results of the studies, from the standard error (SE) and, exceptionally, from the *p*-value [21]. If possible, estimates with more adjusted models were considered (as they are a priori more realistic), considering the highest consumption of dAGEs versus the lowest consumption of dAGEs.

When two or more studies estimated the association between dAGEs intake and cancer risk in the same way (i.e., overall or by specific cancer site) or mortality risk in the same way (i.e., overall or by specific cancer site), random- and fixed-effects meta-analyses were performed [17,22]. Fixed-effects meta-analyses are appropriate for meta-analyses with few studies and statistically non-significant heterogeneity. However, random-effects meta-analyses can be used regardless of the number of studies and heterogeneity. Therefore, whenever heterogeneity allowed, both types of meta-analysis were used to improve the interpretation of the results. The results of the meta-analyses were shown in forest plots. Heterogeneity was assessed using the *I*^2^ statistic, classified as not important if <30%, moderate if 30–50%, substantial if 50–75%, or considerable if >75%, and considered statistically significant if *p* < 0.05 [17]. Publication bias for overall cancer risk, cancer mortality, and other tumours when three or more inputs were included in the meta-analysis, was assessed visually using a funnel plot and the Egger and the Begg tests, considering <0.1 as suggestive of bias [23,24]. Finally, for BC, where available, we considered whether it was progesterone receptor (PR) or oestrogen receptor (ER) positive or negative, and whether it was in situ or invasive.

### 2.7. Sensitivity Analyses

Sensitivity analyses by study exclusion were performed for overall cancer risk, mortality from cancer, and other tumours when three or more inputs were included in the meta-analysis, to test whether any study significantly affected the final estimates of the meta-analysis.

All statistical analyses were conducted with Stata SE software, version 15 (StataCorp, College Station, TX, USA).

## 3. Results

**Figure 1:**
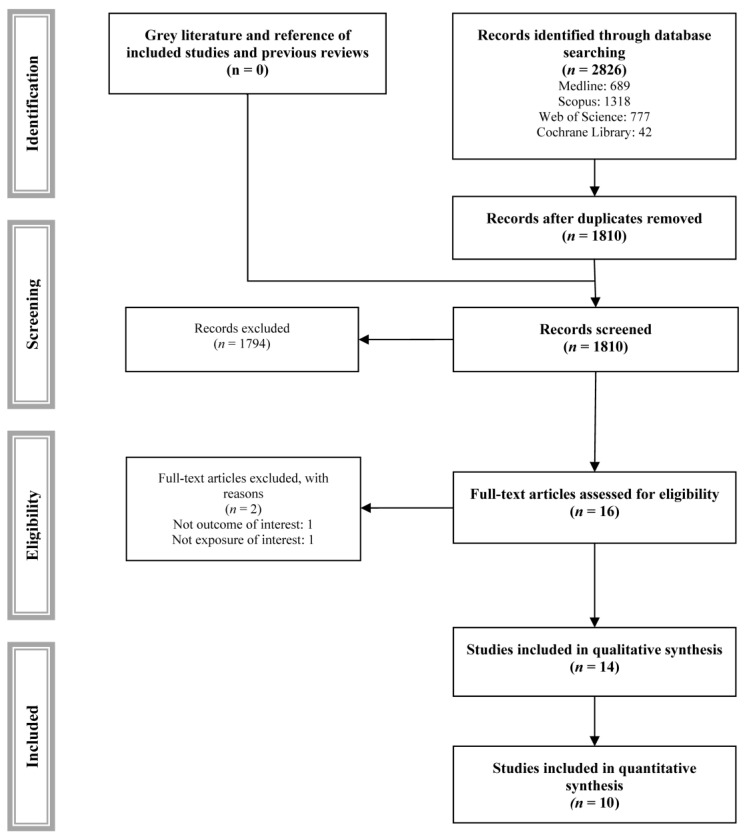
PRISMA flowchart of study selection.

Among the 2726 studies identified, 14 studies met the inclusion and exclusion criteria and were included in the systematic review (Table 1) [25,26,27,28,29,30,31,32,33,34,35,36,37,38], and 10 were included in the meta-analyses. One study [39] was excluded because it estimated the risk of cancer recurrence, and one study [40] because its exposure was serum AGEs (Figure 1).

Studies were conducted in the Americas, Asia, and Europe. All studies were prospective cohorts, mostly derived from observational cohorts, except for two studies that were derived from a trial and two studies that had a case-control design. The samples varied in size from 401 to 528,251 participants, the percentage of females ranged from 41.23 to 100%, and the mean age ranged from 45.6 to 66.3 years. In general, comparisons were made of quartile 4 versus quartile 1, or quintile 5 versus quintile 1. The follow-up of the studies ranged from 4.8 to 27.1 years. Five studies assessed the association between dAGEs and overall cancer, six studies assessed the association between dAGEs and BC, and eight studies assessed the association between dAGEs and other cancers. Finally, most studies used food frequency questionnaires to assess exposure, the International Classification of Diseases for Oncology and the International Classification of Diseases to classify cases, and CML to estimate dAGE intake (Table 1, Supplementary Tables S1 and S2).

### 3.1. Systematic Review

In general, dAGEs intake was not associated with overall cancer risk, except in one study with an HR = 0.99 (95% CI: 0.98, 1.00), nor was it associated with overall cancer mortality. However, it was associated with an increased risk of BC in two studies, with HR = 1.30 (95% CI: 1.04, 1.62) and an OR = 2.33 (95% CI: 1.18, 4.60) (Table 2). According to the stratification of BC by type, high dAGEs intake may increase the risk of PR+, ER+/PR+, in situ and invasive BC, although not all studies reached statistical significance. It may also increase the risk of mortality in all ER and PR subtypes (Supplementary Table S3).

For other types of cancer, the results were inconsistent, with most showing no association, although some studies showed a positive association, particularly for liver cancer with an HR = 2.10 (95% CI: 1.11, 3.98), prostate cancer with an HR = 1.03 (95% CI: 1.01, 1.05), and rectum with an OR = 1.90 (95% CI: 1.14, 3.19); however, three studies reported a negative association, specifically for lung cancer with an HR = 0.95 (95% CI: 0.91, 0.99), rectum cancer with an HR = 0.81 (95% CI: 0.70, 0.93), and stomach cancer with an HR = 0.67 (95% CI: 0.47, 0.96) (Supplementary Table S4).

### 3.2. Risk of Bias Assessment

According to the Study Quality Assessment Tools, all the studies were rated as good. The most affected domain was the lack of blinding of outcome assessors to participants’ exposure or disease status. Other domains affected, but of uncertain relevance, were the lack of justification of sample size to achieve minimum statistical power and the lack of repeated measures of exposure. The assessment of the risk of bias is described in Supplementary Table S5.

### 3.3. Quality of Evidence Assessment

According to the GRADE tool, the association of dAGEs intakes with overall risk of cancer, mortality from cancer, cervical cancer, colon cancer, oesophageal cancer, lung cancer, ovarian cancer, pancreas cancer, prostate cancer, and stomach cancer had a low certainty, while the rest had a very low certainty. The full assessment is available in the Supplementary Table S6.

### 3.4. Meta-Analyses

**Figure 2:**
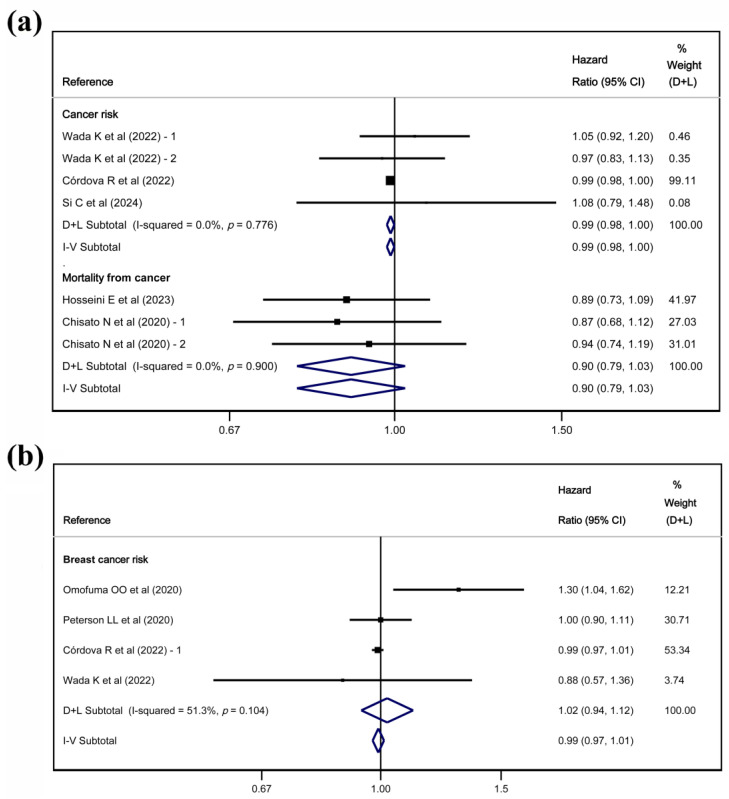
Meta-analyses of the association between dAGEs and overall cancer risk and mortality from cancer (**a**) and meta-analysis of the association between dAGEs and breast cancer risk (**b**) [28,31,32,34,35,36,38].

**Figure 3:**
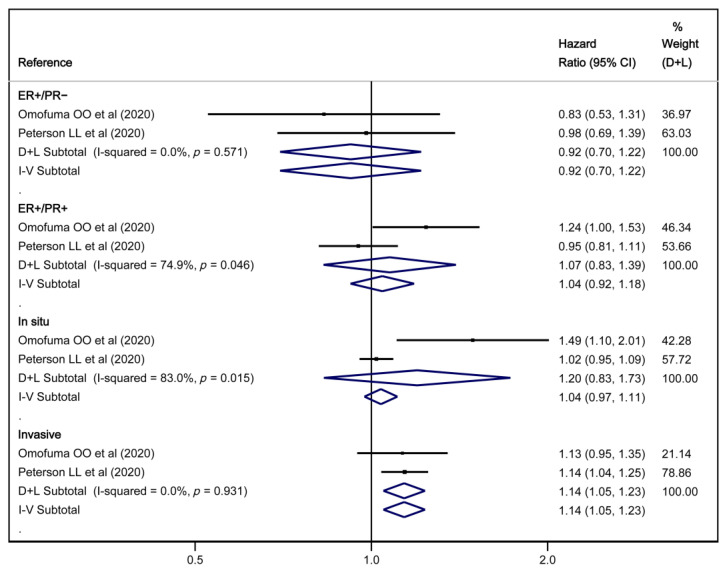
Meta-analysis of the association between dAGEs and breast cancer types [27,28].

The results of the random-effects and fixed-effects meta-analyses showed a reduction in overall cancer risk, with HR = 0.99 (95% CI: 0.98, 1.00). However, it was not associated with either overall cancer mortality or overall BC risk (Figure 2). Regarding the type of BC, high dAGEs intake was associated with a higher risk of invasive cancer, with HR = 1.14 (95% CI: 1.05, 1.23) in both random-effects and fixed-effects models (Figure 3).

**Figure 4:**
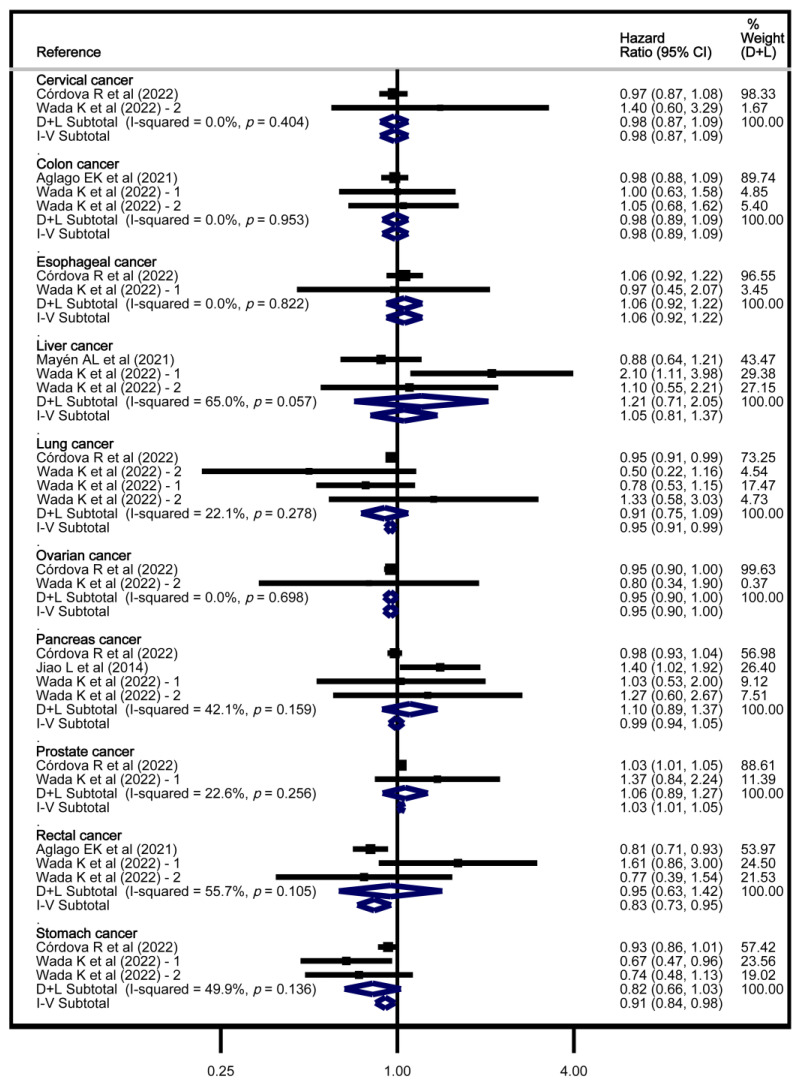
Meta-analysis of the association of dAGEs and cancer risk by localisation [25,29,30,31,32].

Finally, according to the random-effects model, high dAGEs intake was inversely associated with ovarian cancer risk, with HR = 0.95 (95% CI: 0.90, 1.00), whereas according to the fixed effects model, it was positively associated with prostate cancer risk, with HR = 1.03 (95% CI: 1.01, 1.05) and inversely with lung cancer risk with HR = 0.95 (95% CI: 0.91, 0.99), rectum cancer risk with HR = 0.83 (95% CI: 0.73, 0.95), and stomach cancer risk with HR = 0.91 (95% CI: 0.84, 0.98) (Figure 4).

The heterogeneity in the main meta-analyses ranged from not important to substantial, but not statistically significant. There was no evidence of publication bias, either visually or with the Egger and Begg tests (*p* > 0.1) (Figure S1).

### 3.5. Sensitivity Analyses

Sensitivity analyses did not show that any study significantly affected the final estimate for overall cancer risk, although one study showed excessive weight. In addition, the study by Omofuma OO et al. (2020) significantly affected the risk of BC [27], Wada K et al. (2022) (males) affected the risk of lung cancer [32], Jiao L et al. (2014) affected the risk of pancreatic cancer [25], and Wada K et al. (2022) affected the risk of rectum cancer [32]. Sensitivity analyses are described in Figure S2.

## 4. Discussion

### 4.1. Main Findings

To our knowledge, this is the first systematic review and meta-analysis to estimate the association between dAGEs intake and overall cancer risk and mortality, BC, BC subtypes, and other tumour types. Our results showed that a high intake of dAGEs was associated with a slightly lower risk (−1%) of cancer; however, it tended to increase the risk of BC. However, dAGEs intake may influence the risk of certain types of cancer, such as invasive BC, ovarian, prostate, lung cancer, rectum, and stomach.

### 4.2. Findings in Overall Cancer

High dAGEs intake was associated with a 1% lower overall risk of cancer. Despite the statistical significance, a global reduction of 1% may not be clinically significant. Firstly, 1% could be invalid due to possible confounders or covariates not taken into account by the authors, which could slightly alter the estimates obtained (e.g., ethnicity, genetics, etc.). In addition, in both meta-analyses (fixed effects and random effects), the study by Córdova R et al. has more weight because of its narrow confidence intervals, as it is the only study showing a reduction in risk [30]. However, the other studies show no association. In fact, sensitivity analyses excluding the study by Córdova R et al. showed a loss of statistical significance, although the overall estimate from the meta-analysis was not significantly affected. This 1% reduction warrants further investigation; this estimate is likely to be biased by the result of a single study. On the other hand, it is possible that not all cancer cases were included in the estimates, which could also affect the final estimates. In any case, our estimate contrasts with that of the previous review, which found no association or, if anything, an adverse association [14]. This may be because the authors included studies that estimated the individual risk of colorectal, pancreatic, and BC in the meta-analysis of overall cancer risk. This is not necessarily incorrect, but if the effect of AGEs differs according to tumour type, the estimates may differ.

The lack of association, or even the reduction in overall cancer risk, can be partially explained by several mechanisms. Theoretically, AGEs increase oxidative stress and upregulate certain pro-carcinogenic transcription factors (NFkB and STAT3) and other signalling pathways such as the MAPK pathway by binding to the AGE receptor (RAGE) to form the AGE-RAGE complex, which would increase cancer risk. However, AGEs can also bind to other receptors, such as AGER1, found on most cells and tissues, including macrophages, mononuclear cells, and mesangial cells, which accelerates the clearance of AGEs. Similarly, AGER1 inhibits AGE-RAGE and its signalling and reduces oxidative stress and pro-inflammatory cytokines. It should also be noted that the soluble RAGE receptor (sRAGE) can attenuate the effect of AGEs [41,42].

### 4.3. Findings in Breast Cancer

For BC, a high intake of dAGEs was not associated with an overall risk; however, there was an increased risk of BC in subgroup studies. Thus, some studies have shown a dependent association between dAGEs and BC according to the hormone receptors ER and PR, whereas others have not [26,27]. In the meta-analysis, statistical significance was reached in the invasive subtype, with a 14% increased risk. This possible increased risk of BC is supported by the increase in BC when plasma concentrations of AGEs are increased [40]. Regarding the inconsistency of the results according to ER and PR, previous studies have suggested that AGEs are associated with hormone receptor-positive BC [43]. In contrast, other studies have shown that AGEs promote tumour growth in tumours of all ER and PR subtypes, while suppression of RAGE receptors reduces the proliferation of these BC cell lines, regardless of the ER or PR type [44,45,46,47,48].

### 4.4. Findings in Prostate Cancer

Studies of other tumour sites have shown different and sometimes contradictory associations. For example, high dAGEs intake was associated with a 3% increased risk of prostate cancer. With the available evidence, it is not clear whether this increased risk is clinically significant, so further studies are needed to make more precise estimates. However, this possible risk factor is consistent with findings of preclinical experiments and several pathophysiological mechanisms. In prostate cancer cell lines, exposure to CML increased the rate of cell duplication and cell growth and increased the tumour implantation capacity. This could be due to the fact that overexpression of RAGE and its activation by AGEs increases the progression and aggressiveness of this type of cancer by increasing the tumourigenicity of these cells and acquiring cancer stem cell properties. In contrast, the suppression of RAGE reduces this tumourigenicity, which supports this hypothesis [49,50].

### 4.5. Findings in Other Tumour Sites

Interestingly, the fixed meta-analyses showed an inverse association of dAGEs consumption with lung and ovarian cancer, with a −5% risk in each case, with stomach cancer, with a −9% risk, and with rectal cancer, with a −17% risk. These estimates should be treated with caution for several reasons: firstly, because of the small number of studies included; secondly, because some studies showed no association or a non-statistically significant trend towards a harmful association; and finally, because unrecognised biases cannot be ruled out. For example, the association between dAGEs and serum AGEs is unclear, which could lead to spurious associations, either positive or negative. This would disassociate the effect of dAGEs on cancer and focus attention on other nutrients with potentially beneficial or harmful effects [51]. Nevertheless, these are interesting results, and, in some cases, they can be explained. For example, in lung cancer, RAGE is highly expressed in lung tissue, but its expression is reduced in lung cancer. In animal models, re-expression of RAGE reduces the growth of the tumour [52,53]. In gastric cancer, although RAGE is overexpressed, CML causes the production of melanoidins, which have antioxidant properties, metal chelation, and inhibition of lipid peroxidation, particularly in the gastrointestinal tract, in addition to counteracting RAGE overexpression. Melanoidins may also inhibit Helicobacter pylori colonisation [54,55,56,57,58,59]. It is also important to note that not all sources of dAGEs, such as CML, come from unhealthy foods; there are foods rich in CML that are healthy in themselves, such as nuts or legumes, which may reduce the risk of cancer and other diseases, such as cardiovascular disease [34,60].

### 4.6. Implications

This systematic review and meta-analysis has several interrelated clinical and research implications. First, dAGEs consumption may differentially affect the risk of cancer in certain tumours. The fact that the risk of development or progression increases or decreases depending on the type of tumour may be useful in developing therapeutic strategies. Second, since dAGEs intake comes from different foods, some of which have beneficial properties, it is not possible to make a priori recommendations to increase or decrease AGE intake. In fact, on the basis of the available evidence, it is of greater interest to adopt a healthy lifestyle, including a healthy diet and a good cooking technique, regardless of AGE consumption, the effect of which is unclear.

### 4.7. Limitations

This systematic review and meta-analysis has several limitations that should be considered. First, although the sample sizes were large, further studies are needed to increase the statistical power of the meta-analyses reviewed. Second, most studies used the CML as an indicator of dAGEs, and when we had a choice, we chose the CML. However, firstly, the CML is one of the possible dAGEs, and secondly, the estimates are generally obtained from questionnaires, which may introduce some bias. Third, we always compared the participants who consumed the most AGEs with those who consumed the least, but each population consumes different average amounts of AGEs. In addition, some studies used quintiles, other quartiles, and other tertiles. Fourth, most studies are based on previous studies of dAGEs in foods, but foods may vary in their dAGE content depending on the food quality and cooking method used in the population in which the database was generated. Therefore, it may be necessary to update the database. Fifth, in the meta-analyses, consistency was maintained by separating studies that studied overall cancer risk from those that included specific cancers. However, in the systematic review, although the distinction between specific outcomes was maintained, the wide variety of outcomes made interpretation more complex because of this heterogeneity. Sixth, there is a limitation of studies by cancer site, which impedes the performance of meta-analysis or the interpretation of the findings in the systematic review, which limits the scope of our study. Seventh, the use of self-reported questionnaires could lead to recall bias. In addition, the inclusion of several studies on breast cancer, which mainly affects women, could be another bias in the systematic review compared to the analysis of all studies as a whole, because it involves the inclusion of a higher percentage of women. And eighth, the certainty of all outcomes was from low to very low.

## 5. Conclusions

This systematic review and meta-analysis suggested that dAGEs do not increase the overall risk of cancer. However, their potential effect varies depending on the type of tumour, with an increased risk for prostate cancer and a possible decreased risk for lung, ovarian, stomach, and rectum cancers. On the basis of the available evidence, the importance of a healthy diet should prevail, regardless of the consumption of dAGEs. Finally, knowledge of the impact of AGEs on the development or prevention of cancer could open up new lines of research into the treatment of these diseases.

## Figures and Tables

**Table 1:** Baseline characteristics of participants in the included studies.

Reference	Region	Design	Sample Size	Females (%)	Age (years)	Comparison	Length (years)	Outcome		
								Total	BC	Others
Cancer risk										
Jiao L et al. (2014) [25]	US	Cohorts (Prospective)	528,251	41.23	62.1 ± 5.4	Quintile 5 vs. Quintile 1	10.5	-	-	✓
Kong SY et al. (2015) [26]	Denmark, France, Greece, Germany, Italy, Netherlands, Norway, Spain, Sweden, UK	Case-control	2110	51.7	58.5	Quartile 4 vs. Quartile 1	-	-	-	✓
Omofuma OO et al. (2020)—1 [27]	US	Cohorts (Prospective)	27,464	100	62.4	Quintile 5 vs. Quintile 1	11.5	-	✓	-
Peterson LL et al. (2020) [28]	US	Cohorts (Prospective)	183,548	100	61.6	Quintile 5 vs. Quintile 1	12.8	-	✓	-
Aglago EK et al. (2021) [29]	Europe	Cohorts (Prospective)	450,111	70.80	50.6	Quintile 5 vs. Quintile 1	14.1	-	-	✓
Mayén AL et al. (2021) [30]	Europe	Cohorts (Prospective)	450,111	70.80	50.6	Tertile 3 vs. Tertile 1	14.9	-	-	✓
Córdova R et al. (2022) [31]	Europe	Cohorts (Prospective)	450,111	70.80	50.6	Quintile 5 vs. Quintile 1	14.9	✓	✓	✓
Wada K et al. (2022) [32]	Japan	Cohorts (Prospective)	30,722	53.87	M: 55.1 F: 56.3	Quartile 4 vs. Quartile 1	-	✓	✓	✓
Jahromi MK et al. (2023) [33]	Iran	Case-control	401	100	47.9 ± 10.3	Tertile 3 vs. Tertile 1	-	-	✓	-
Si C et al. (2024) [34]	US	Cohorts (Prospective)	22,124	57.5	45.6 ± 0.4	Quartile 4 vs. Quartile 1	27.1	✓	-	-
Mortality										
Chisato N et al. (2020) [35]	Japan	Cohorts	29,079	54.07	54.56	Quartile 4 vs. Quartile 1	14.1	✓	-	-
Omofuma OO et al. (2020)—2 [36]	US	Cohorts	2023	100	-	Tertile 3 vs. Tertile 1	15.1	-	✓	-
Mao Z et al. (2021) [37]	Europe	Cohorts	5801	57.2	66.3	Quintile 5 vs. Quintile 1	4.8	-	-	✓
Hosseini E et al. (2023) [38]	Iran	Cohorts	48,632	57.52	52.0 ± 8.9	Quintile 5 vs. Quintile 1	13.5	✓	-	✓

Abbreviations: UK—United Kingdom; US—United States; BC—Breast Cancer.

**Table 2:** Main results of the systematic review.

Reference	Comparison	Type of Association	Overall Cancer (95% CI)	Breast Cancer (95% CI)
Cancer risk				
Omofuma OO et al. (2020)—1 [27]	Q5 vs. Q1	Hazard Ratio	-	1.30 (1.04, 1.62) *
Peterson LL et al. (2020) [28]	Q5 vs. Q1	Hazard Ratio	-	1.00 (0.90, 1.11)
Córdova R et al. (2022) [31]	Q5 vs. Q1	Hazard Ratio	0.99 (0.98, 1.00) *	0.99 (0.97, 1.01)
Wada K et al. (2022) [32]	Q4 vs. Q1—males	Hazard Ratio	1.05 (0.92, 1.20	-
Wada K et al. (2022) [32]	Q4 vs. Q1—females	Hazard Ratio	0.97 (0.83, 1.13)	0.88 (0.57, 1.36)
Jahromi MK et al. (2023) [33]	T3 vs. T1	Odds Ratio	-	2.33 (1.18, 4.60) *
Si C et al. (2024) [34]	Q4 vs. Q1	Hazard Ratio	1.08 (0.78, 1.48)	-
Mortality				
Chisato N et al. (2020)—1 [35]	Q4 vs. Q1—males	Hazard Ratio	0.87 (0.67, 1.12)	-
Chisato N et al. (2020)—2 [35]	Q4 vs. Q1—females	Hazard Ratio	0.94 (0.74, 1.19)	-
Omofuma OO et al. (2020)—2 [36]	T3 vs. T1	Hazard Ratio	-	1.49 (0.98–2.24)
Hosseini E et al. (2023) [38]	Q5 vs. Q1	Hazard Ratio	0.89 (0.76, 1.03)	-

Abbreviations: Q5 vs. Q1—Quintile 5 vs. Quintile 1; Q4 vs. Q1—Quartile 4 vs. Quartile 1; T3 vs. T1—Tertile 3 vs. Tertile 1; * indicates *p* < 0.05.

## Data Availability

Data are available upon reasonable request to the corresponding author.

## Supplementary Materials

The following supporting information can be downloaded at: https://www.mdpi.com/article/10.3390/nu17101638/s1, Table S1. Exposure measurement and case criteria; Table S2. Dietary AGEs intake in the included comparisons; Table S3. Results in breast cancer by characteristics; Table S4. Results in other cancers; Table S5. Risk of bias assessment; Table S6. Quality of evidence assessment; Figure S1. Publication bias assessment by visually inspection with funnel plots; Figure S2. Sensitivity analyses; Appendix S1. Search strategy.

## Abbreviations

The following abbreviations are used in this manuscript:

95% CI	95% confidence intervals
AGEs	Advanced glycation end products
BC	Breast Cancer
CML	N-ε-[carboxymethyl]-L-lysine
dAGEs	Dietary advanced glycation end products
GRADE	Grading of Recommendations, Assessment, Development and Evaluation
MH-H1	N-δ-[5-hydro-5-methyl-4-imidazolon-2-yl]-ornithine
MOOSE	Meta-analysis of Observational Studies in Epidemiology
PRISMA	Preferred Reporting Items for Systematic Reviews and Meta-Analyses
RAGE	Receptor for advanced glycation end products
SE	Standard error
