# Supplementary material for: Association of Dietary Advanced Glycation End Products with Overall and Site-Specific Cancer Risk and Mortality: A Systematic Review and Meta-Analysis
Source: Nutrients. 2025 May 10;17(10):1638. doi: 10.3390/nu17101638 (PMC12113765; doi:10.3390/nu17101638)
Supplement: Supplementary file 1 [file nutrients-17-01638-s001.zip › nutrients-3624709-supplementary.pdf]

SUPPLEMENTARY MATERIAL

Association of dietary advanced glycation end products with overall and site-specific cancer risk and mortality: a systematic review and meta-analysis

Nutrients

Carlos Pascual-Morena, Miriam Garrido-Miguel, Irene Martínez-García, Maribel Lucerón-Lucas-Torres, Eva Rodríguez-Gutiérrez, Carlos Berlanga-Macías, Jaime Fernández-Bravo-Rodrigo, Silvana Patiño-Cardona

Correspondence: Irene Martínez-García, CarVasCare Research Group, Facultad Enfermería de Cuenca, Universidad de Castilla-La Mancha, Spain (irene.mgarcia@uclm.es).

INDEX

Table S1. Exposure measurement and cases criteria.

Table S2. Dietary AGEs intake in the included comparisons.

Table S3. Results in breast cancer by characteristics.

Table S4. Results in other cancers.

Table S5. Risk of bias assessment.

Table S6. Quality of evidence assessment.

Figure S1. Publication bias assessment by visually inspection with funnelplots.

Figure S2. Sensitivity analyses.

Appendix S1. Search strategy.

Table S1. Exposure measurement and cases criteria.

Reference	Exposure	Cancer cases
Jiao L et al (2015)	Food-Frequency Questionnaire	International Classification of Diseases for Oncology, Third Edition
Kong SY et al (2015)	Food-Frequency Questionnaire	International Statistical Classification of Diseases, Injury, and Cause of Death
Omofuma OO et al (2020)	Food-Frequency Questionnaire	Self-reported, registers
Peterson LL et al (2020)	Food-Frequency Questionnaire	International Classification of Diseases for Oncology, Third Edition
Aglago EK et al (2021)	Food-Frequency Questionnaire and others	International Classification of Diseases for Oncology
Mayén AL et al (2021)	Food-Frequency Questionnaire and others	International Statistical Classification of Diseases, Injuries and Causes of Death and the International Classification of Diseases for Oncology
Córdova R et al (2022)	Food-Frequency Questionnaire and others	International Statistical Classification of Diseases, Injuries and Causes of Death and the International Classification of Diseases for Oncology
Wada K et al (2022)	Food-Frequency Questionnaire	International Classification of Diseases and Health Related Problem
Jahromi MK et al (2023)	Food-Frequency Questionnaire	Questionnaire
Si C et al (2024)	Food-Frequency Questionnaire	International Statistical Classification of Diseases
Nagata C et al (2020)	Food-Frequency Questionnaire	International Classification of Diseases
Omofuma OO et al (2021)	Food-Frequency Questionnaire	Registers
Mao Z et al (2021)	Food-Frequency Questionnaire and others	International Classification of Disease and the International Classification of Diseases for Oncology
Hosseini E et al (2023)	Food-Frequency Questionnaire	Questionnaire

Table S2. Dietary AGEs intake in the included comparisons.

Reference	Type of AGE	Comparison	Lowest	Highest
Jiao L et al (2014)	CML	Q5 vs Q1	50–6866 KU/1000 Kcal/day	17261–101909 KU/1000 Kcal/day
Kong SY et al (2015)	Glycer-AGEs	Q4 vs Q1	4.3 U/mL/day	6.3 U/mL/day
Omofuma OO et al (2020) – 1	CML	Q5 vs Q1	< 4057 KU/1000 Kcal/day	>7732 KU/1000 Kcal/day
Peterson LL et al (2020)	Likely CML	Q5 vs Q1	3555 KU/1000 Kcal/day	8644 KU/1000 Kcal/day
Aglago EK et al (2021)	CML	Q5 vs Q1	0.92 mg/day	0.98 mg/day
Mayén AL et al (2021)	CML	T3 vs T1	1.0 mg/day	1.3 mg/day
Córdova R et al (2022)	CML	Q5 vs Q1	2.1 mg/day	4.3 mg/day
Wada K et al (2022)	CML	Q4 vs Q1	1.73 mg/day	3.48 mg/day
Jahromi MK et al (2023)	Likely CML	T3 vs T1	6594 KU/day	13628 KU/day
Si C et al (2024)	CML	Q4 vs Q1	Score: 0–12,67	Score: 39,21–114,61
Chisato N et al (2020) – 1	CML	Q4 vs Q1 – males	2.41 mg/day	3.35 mg/day
Chisato N et al (2020) – 2	CML	Q4 vs Q1 – females	2.50 mg/day	3.62 mg/day
Omofuma OO et al (2020)	CML	T3 vs T1	<5549 KU/1000 Kcal/day	>7311 KU/1000 Kcal/day
Mao Z et al (2021)	CML	Q5 vs Q1	<2.3 mg/day	>3.7 mg/day
Hosseini E et al (2023)	CML	Q5 vs Q1	-	-

Abbreviations: Q5 vs Q1 – Quintile 5 vs Quintile 1; Q4 vs Q1 – Quartile 4 vs Quartile 1; T3 vs T1 – Tertile 3 vs Tertile 1; KU – kilounits; Kcal – Kilocalories; ml – millilitre; mg – milligrams; * indicates $p < 0.05$

Table S3. Results in breast cancer by characteristics.

Reference	Comparison	Type of association	Characteristics	Association (95%CI)
Omofuma OO et al (2020) – 1	Q5 vs Q1	Hazard Ratio	ER-	1.21 (0.82, 1.79)
Omofuma OO et al (2020) – 1	Q5 vs Q1	Hazard Ratio	ER+	1.18 (0.97, 1.45)
Omofuma OO et al (2020) – 1	Q5 vs Q1	Hazard Ratio	PR-	1.04 (0.76, 1.41)
Omofuma OO et al (2020) – 1	Q5 vs Q1	Hazard Ratio	PR+	1.24 (1.01, 1.53)*
Peterson LL et al (2020)	Q5 vs Q1	Hazard Ratio	ER-/PR-	0.94 (0.68, 1.30)
Omofuma OO et al (2020) – 1	Q5 vs Q1	Hazard Ratio	ER+/PR-	0.83 (0.53, 1.31)
Peterson LL et al (2020)	Q5 vs Q1	Hazard Ratio	ER+/PR-	0.98 (0.70, 1.39)
Omofuma OO et al (2020) – 1	Q5 vs Q1	Hazard Ratio	ER+/PR+	1.24 (1.01, 1.53)*
Peterson LL et al (2020)	Q5 vs Q1	Hazard Ratio	ER+/PR+	0.95 (0.82, 1.11)
Omofuma OO et al (2020) – 1	Q5 vs Q1	Hazard Ratio	In situ	1.49 (1.11, 2.01)*
Peterson LL et al (2020)	Q5 vs Q1	Hazard Ratio	In situ	1.02 (0.96, 1.09)
Omofuma OO et al (2020) – 1	Q5 vs Q1	Hazard Ratio	Invasive	1.13 (0.94, 1.35)
Peterson LL et al (2020)	Q5 vs Q1	Hazard Ratio	Invasive	1.14 (1.04, 1.25)*
**Mortality**				
Omofuma OO et al (2020) – 2	T3 vs T1	Hazard Ratio	ER-	1.92 (1.01, 3.62)*
Omofuma OO et al (2020) – 2	T3 vs T1	Hazard Ratio	ER+	1.36 (1.04, 1.80)*
Omofuma OO et al (2020) – 2	T3 vs T1	Hazard Ratio	PR-	1.79 (1.10, 2.92)*
Omofuma OO et al (2020) – 2	T3 vs T1	Hazard Ratio	PR+	(0.97, 1.78)
Omofuma OO et al (2020) – 2	T3 vs T1	Hazard Ratio	ER-/PR-	2.02 (1.01, 4.05)*
Omofuma OO et al (2020) – 2	T3 vs T1	Hazard Ratio	ER+/PR+	1.37 (1.05, 1.80)*

Abbreviations: Q5 vs Q1 – Quintile 5 vs Quintile 1; T3 vs T1 – Tertile 3 vs Tertile 1; * indicates $p < 0.05$

Table S4. Results in other cancers.

Reference	Comparison	Type	Localisation of the cancer	Association (95%CI)
**Cancer risk**				
Wada K et al (2022)	Q4 vs Q1	Hazard Ratio	Bladder	0.84 (0.49, 1.45)
Córdova R et al (2022)	Q5 vs Q1	Hazard Ratio	Cervical	0.97 (0.87, 1.08)
Wada K et al (2022)	Q4 vs Q1	Hazard Ratio	Cervical	1.40 (0.60, 3.29)
Kong SY et al (2015)	Q4 vs Q1	Odds Ratio	Colon	0.83 (0.57, 1.22)
Aglago EK et al (2021)	Q5 vs Q1	Hazard Ratio	Colon	0.98 (0.89, 1.09)
Wada K et al (2022)	Q4 vs Q1 – males	Hazard Ratio	Colon	1.00 (0.63, 1.58)
Wada K et al (2022)	Q4 vs Q1 – females	Hazard Ratio	Colon	1.05 (0.68, 1.62)
Kong SY et al (2015)	Q4 vs Q1	Odds Ratio	Colorectum	1.10 (0.82, 1.49)
Wada K et al (2022)	Q4 vs Q1	Hazard Ratio	Endometrial	1.74 (0.65, 4.66)
Mayén AL et al (2021)	T3 vs T1	Hazard Ratio	Liver	0.88 (0.63, 1.21)
Wada K et al (2022)	Q4 vs Q1 – males	Hazard Ratio	Liver	2.10 (1.11, 3.98)*
Wada K et al (2022)	Q4 vs Q1 – females	Hazard Ratio	Liver	1.10 (0.55, 2.21)
Córdova R et al (2022)	Q5 vs Q1	Hazard Ratio	Lung	0.95 (0.91, 0.99)*
Wada K et al (2022)	Q4 vs Q1 – males	Hazard Ratio	Lung (ever smokers)	0.78 (0.54, 1.15)
Wada K et al (2022)	Q4 vs Q1 – females	Hazard Ratio	Lung (ever smokers)	1.33 (0.58, 3.03)
Wada K et al (2022)	Q4 vs Q1 – females	Hazard Ratio	Lung (never smokers)	0.50 (0.21, 1.16)
Córdova R et al (2022)	Q5 vs Q1	Hazard Ratio	Oesophageal	1.06 (0.92, 1.22)
Wada K et al (2022)	Q4 vs Q1	Hazard Ratio	Oesophageal	0.97 (0.45, 2.07)
Córdova R et al (2022)	Q5 vs Q1	Hazard Ratio	Ovarian	0.95 (0.90, 1.00)
Wada K et al (2022)	Q4 vs Q1	Hazard Ratio	Ovarian	0.80 (0.34, 1.90)
Córdova R et al (2022)	Q5 vs Q1	Hazard Ratio	Pancreas	0.98 (0.92, 1.04)
Jiao L et al (2014)	Q5 vs Q1	Hazard Ratio	Pancreas	1.40 (1.02, 1.92)
Wada K et al (2022)	Q4 vs Q1 – males	Hazard Ratio	Pancreas	1.03 (0.53, 2.00)
Wada K et al (2022)	Q4 vs Q1 – females	Hazard Ratio	Pancreas	1.27 (0.60, 2.67)
Córdova R et al (2022)	Q5 vs Q1	Hazard Ratio	Prostate	1.03 (1.01, 1.05)*
Wada K et al (2022)	Q4 vs Q1	Hazard Ratio	Prostate	1.37 (0.84, 2.24)
Kong SY et al (2015)	Q4 vs Q1	Odds Ratio	Rectum	1.90 (1.14, 3.19)*
Aglago EK et al (2021)	Q5 vs Q1	Hazard Ratio	Rectum	0.81 (0.70, 0.93)*
Wada K et al (2022)	Q4 vs Q1 – males	Hazard Ratio	Rectum	1.61 (0.86, 3.00)
Wada K et al (2022)	Q4 vs Q1 – females	Hazard Ratio	Rectum	0.77 (0.39, 1.54)
Córdova R et al (2022)	Q5 vs Q1	Hazard Ratio	Stomach	0.93 (0.85, 1.01)
Wada K et al (2022)	Q4 vs Q1 – males	Hazard Ratio	Stomach	0.67 (0.47, 0.96)*
Wada K et al (2022)	Q4 vs Q1 – females	Hazard Ratio	Stomach	0.74 (0.48. 1.13)
**Mortality**				
Mao Z et al (2021)	Q5 vs Q1	Hazard Ratio	Colorectum	1.16 (0.98, 1.36)
Hosseini E et al (2023)	Q5 vs Q1	Hazard Ratio	Gastrointestinal	0.89 (0.72, 1.09)

Abbreviations: Q5 vs Q1 – Quintile 5 vs Quintile 1; Q4 vs Q1 – Quartile 4 vs Quartile 1; T3 vs T1 – Tertile 3 vs Tertile 1; * indicates $p < 0.05$

Table S5. Risk of bias assessment.

a) Cohorts' studies.

Reference	1	2	3	4	5	6	7	8	9	10	11	12	13	14	15
Jiao L et al (2014)	Green	Green	Green	Green	Yellow	Green	Green	Green	Green	Green	Green	Red	Green	Green	Green
Omofuma OO et al (2020) – 1	Green	Green	Green	Green	Yellow	Green	Green	Green	Green	Green	Green	Red	Green	Green	Green
Peterson LL et al (2020)	Green	Green	Green	Green	Yellow	Green	Green	Green	Green	Green	Green	Red	Green	Green	Green
Aglago EK et al (2021)	Green	Green	Green	Green	Yellow	Green	Green	Green	Green	Yellow	Green	Red	Green	Green	Green
Mayén AL et al (2021)	Green	Green	Green	Green	Yellow	Green	Green	Green	Green	Yellow	Green	Red	Green	Green	Green
Córdova R et al (2022)	Green	Green	Green	Green	Yellow	Green	Green	Green	Green	Yellow	Green	Red	Green	Green	Green
Wada K et al (2022)	Green	Green	Green	Green	Yellow	Green	Green	Green	Green	Yellow	Green	Red	Green	Green	Green
Si C et al (2024)	Green	Green	Green	Green	Yellow	Green	Green	Green	Green	Yellow	Green	Red	Green	Green	Green
Chisato N et al (2020)	Green	Green	Green	Green	Yellow	Green	Green	Green	Green	Yellow	Green	Red	Green	Green	Green
Omofuma OO et al (2020) – 2	Green	Green	Green	Green	Yellow	Green	Green	Green	Green	Green	Green	Red	Green	Green	Green
Mao Z et al (2021)	Green	Green	Green	Green	Yellow	Green	Green	Green	Green	Yellow	Green	Red	Green	Green	Green
Hosseini E et al (2023)	Green	Green	Green	Green	Yellow	Green	Green	Green	Green	Yellow	Green	Red	Green	Green	Green

Assessment of risk of bias for observational studies with 'Study Quality Assessment Tools'. Green: good/low risk; Red: poor/high risk; Yellow: fair/some concerns/not available/not applicable

Items for Study Quality Assessment Tools:

1. Was the research question or objective in this paper clearly stated?
2. Was the study population clearly specified and defined?
3. Was the participation rate of eligible persons at least 50%?
4. Were all the subjects selected or recruited from the same or similar populations (including the same time period)? Were inclusion and exclusion criteria for being in the study prespecified and applied uniformly to all participants?
5. Was a sample size justification, power description, or variance and effect estimates provided?
6. For the analyses in this paper, were the exposure(s) of interest measured prior to the outcome(s) being measured?
7. Was the timeframe sufficient so that one could reasonably expect to see an association between exposure and outcome if it existed?
8. For exposures that can vary in amount or level, did the study examine different levels of the exposure as related to the outcome (e.g., categories of exposure, or exposure measured as continuous variable)?
9. Were the exposure measures (independent variables) clearly defined, valid, reliable, and implemented consistently across all study participants?
10. Was the exposure(s) assessed more than once over time?
11. Were the outcome measures (dependent variables) clearly defined, valid, reliable, and implemented consistently across all study participants?
12. Were the outcome assessors blinded to the exposure status of participants?
13. Was loss to follow-up after baseline 20% or less?
14. Were key potential confounding variables measured and adjusted statistically for their impact on the relationship between exposure(s) and outcome(s)?
15. Overall bias: good, fair, or poor. If poor, it need a justification.

b) Case-controls' studies.

**Reference**	**1**	**2**	**3**	**4**	**5**	**6**	**7**	**8**	**9**	**10**	**11**	**12**	**13**
Kong SY et al (2015)													
Jahromi MK et al (2023)													

Assessment of risk of bias for case-control studies with 'Study Quality Assessment Tools'. Green: good/low risk; Red: poor/high risk; Yellow: fair/some concerns/not available/not applicable

Items for Study Quality Assessment Tools:

1. Was the research question or objective in this paper clearly stated and appropriate?
2. Was the study population clearly specified and defined?
3. Did the authors include a sample size justification?
4. Were controls selected or recruited from the same or similar population that gave rise to the cases (including the same timeframe)?
5. Were the definitions, inclusion and exclusion criteria, algorithms or processes used to identify or select cases and controls valid, reliable, and implemented consistently across all study participants?
6. Were the cases clearly defined and differentiated from controls?
7. If less than 100 percent of eligible cases and/or controls were selected for the study, were the cases and/or controls randomly selected from those eligible?
8. Was there use of concurrent controls?
9. Were the investigators able to confirm that the exposure/risk occurred prior to the development of the condition or event that defined a participant as a case?
10. Were the measures of exposure/risk clearly defined, valid, reliable, and implemented consistently (including the same time period) across all study participants?
11. Were the assessors of exposure/risk blinded to the case or control status of participants?
12. Were key potential confounding variables measured and adjusted statistically in the analyses? If matching was used, did the investigators account for matching during study analysis?

Table S6. Quality of evidence assessment.

Certainty assessment							Effect Size	Certainty
**№ of studies**	**Study design**	**Risk of bias**	**Inconsistency**	**Indirectness**	**Imprecision**	**Other considerations**		
**A. Cancer risk**								
**4**	Observational	Non-serious	Non-serious	Non-serious	Non-serious	None	Random: HR = 0.99 (0.98, 1.00) Fixed: HR = 0.99 (0.98, 1.00)	Low
**B. Mortality**								
**3**	Observational	Non-serious	Non-serious	Non-serious	Non-serious	None	Random: HR = 0.90 (0.79, 1.03) Fixed: HR = 0.90 (0.79, 1.03)	Low
**C. Breast cancer risk**								
**4**	Observational	Non-serious	Serious	Non-serious	Non-serious	None	Random: HR = 1.02 (0.94, 1.12) Fixed: HR = 0.99 (0.97, 1.01)	Very low
**D. Cervical cancer**								
**2**	Observational	Non-serious	Non-serious	Non-serious	Non-serious	None	Random: HR = 0.98 (0.87, 1.09) Fixed: HR = 0.98 (0.87, 1.09)	Low
**E. Colon cancer**								
**3**	Observational	Non-serious	Non-serious	Non-serious	Non-serious	None	Random: HR = 0.98 (0.89, 1.09) Fixed: HR = 0.98 (0.89, 1.09)	Low
**F. Oesophageal cancer**								
**2**	Observational	Non-serious	Non-serious	Non-serious	Non-serious	None	Random: HR = 1.06 (0.92, 1.22) Fixed: HR = 1.06 (0.92, 1.22)	Low
**G. Liver cancer**								
**3**	Observational	Non-serious	Serious	Non-serious	Serious	None	Random: HR = 1.21 (0.71, 2.05) Fixed: HR = 1.05 (0.81, 1.37)	Very low
**H. Lung cancer**								
**4**	Observational	Non-serious	Non-serious	Non-serious	Non-serious	None	Random: HR = 0.91 (0.75, 1.09) Fixed: HR = 0.95 (0.91, 0.99)	Low
**I. Ovarian cancer**								
**2**	Observational	Non-serious	Non-serious	Non-serious	Non-serious	None	Random: HR = 0.95 (0.90, 1.00) Fixed: HR = 0.95 (0.90, 1.00)	Low
**J. Pancreas cancer**								
**4**	Observational	Non-serious	Non-serious	Non-serious	Non-serious	None	Random: HR = 1.10 (0.89, 1.37)	Low

							Fixed: HR = 0.99 (0.94, 1.05)	
**K.**	**Prostate cancer**							
**2**	Observational	Non-serious	Non-serious	Non-serious	Non-serious	None	Random: HR = 1.06 (0.89, 1.27) Fixed: HR = 1.03 (1.01, 1.05)	Low
**L.**	**Rectal cancer**							
**3**	Observational	Non-serious	Serious	Non-serious	Serious	None	Random: HR = 0.95 (0.63, 1.42) Fixed: HR = 0.83 (0.73, 0.95)	Very low
**M.**	**Stomach cancer**							
**3**	Observational	Non-serious	Non-serious	Non-serious	Non-serious	None	Random: HR = 0.82 (0.66, 1.03) Fixed: HR = 0.91 (0.84, 0.98)	Low

Figure S1. Publication bias assessment by visually inspection with funnelplots.

A. Overall cancer risk.

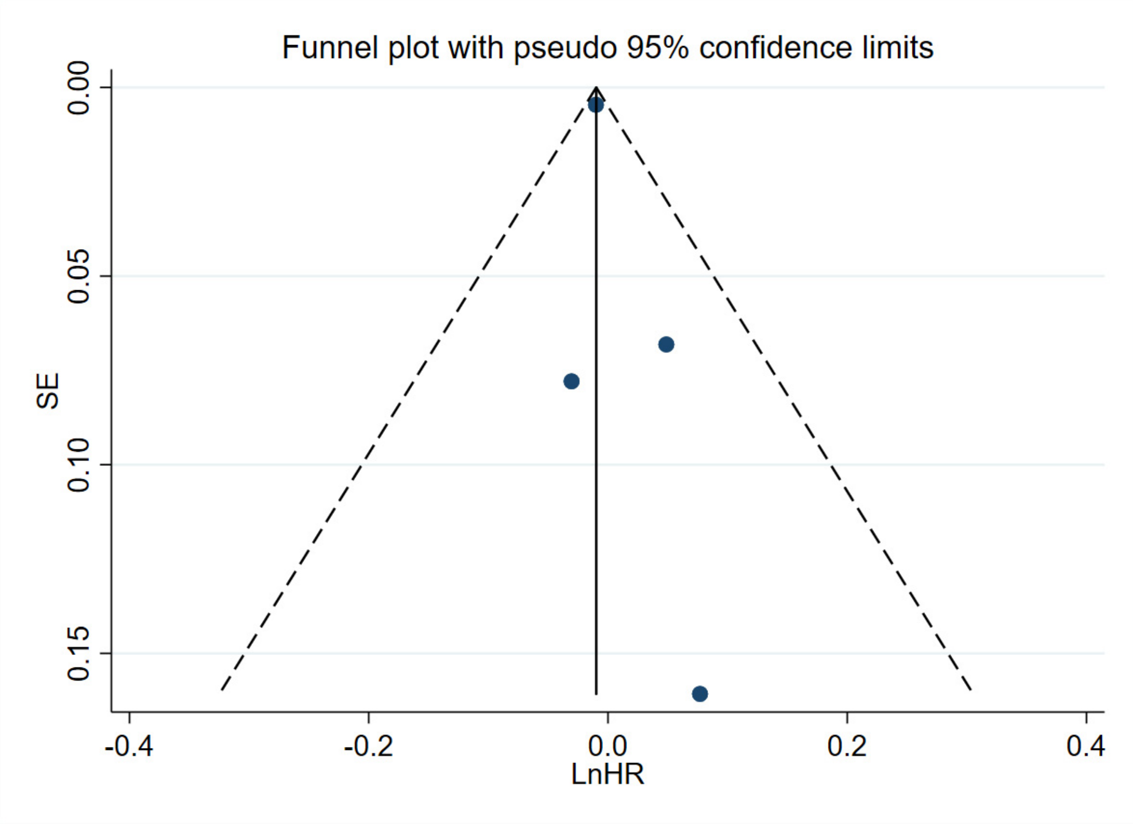

B. Cancer mortality.

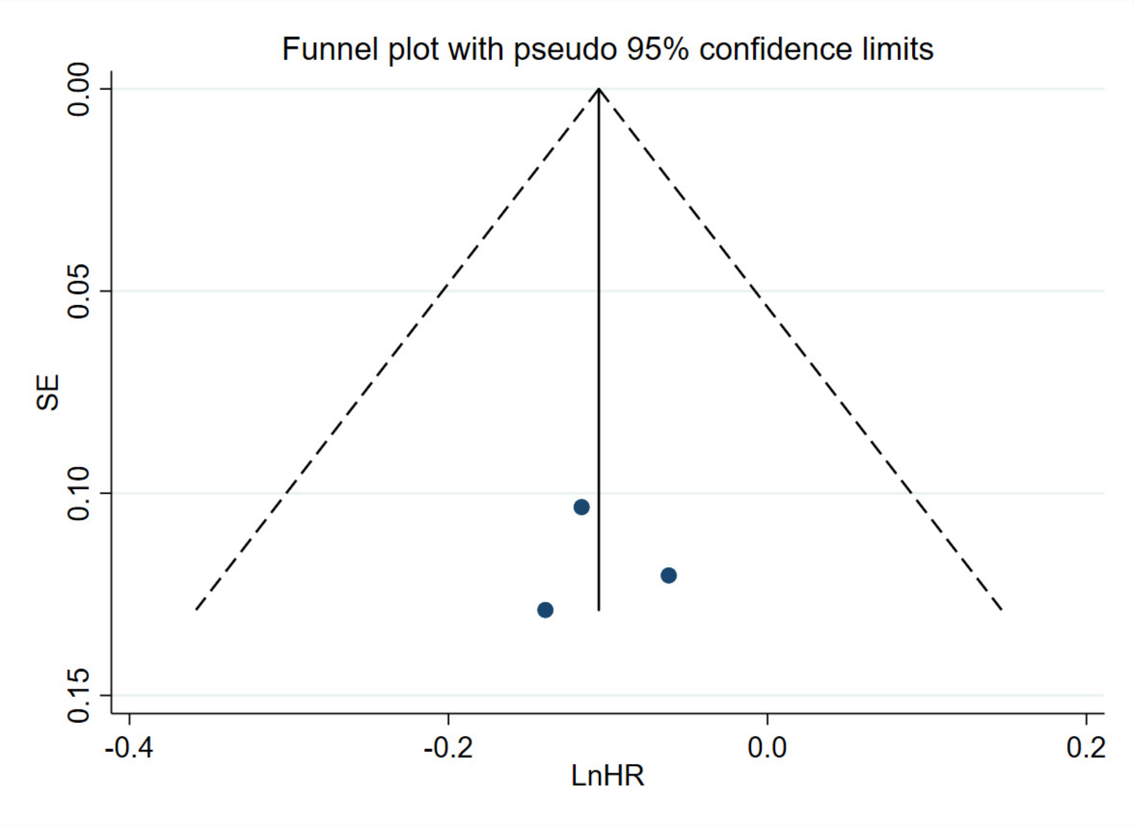

C. Breast cancer risk.

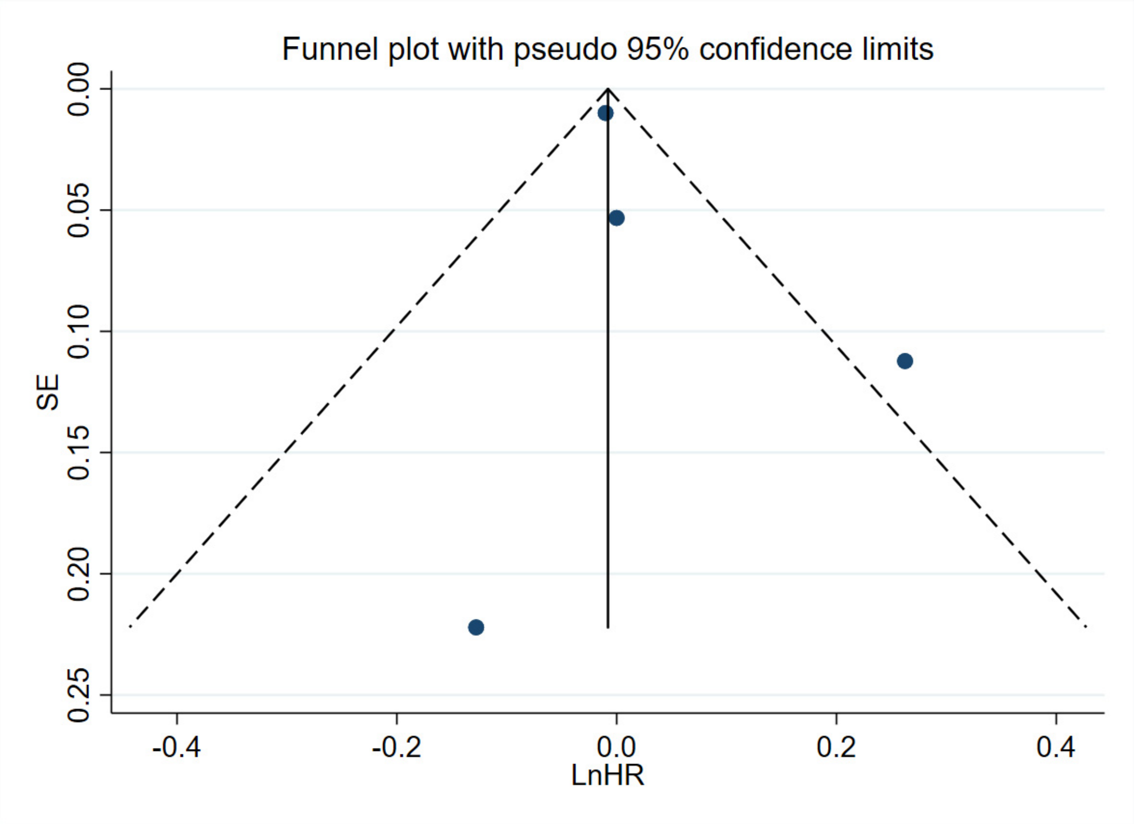

D. Colon cancer.

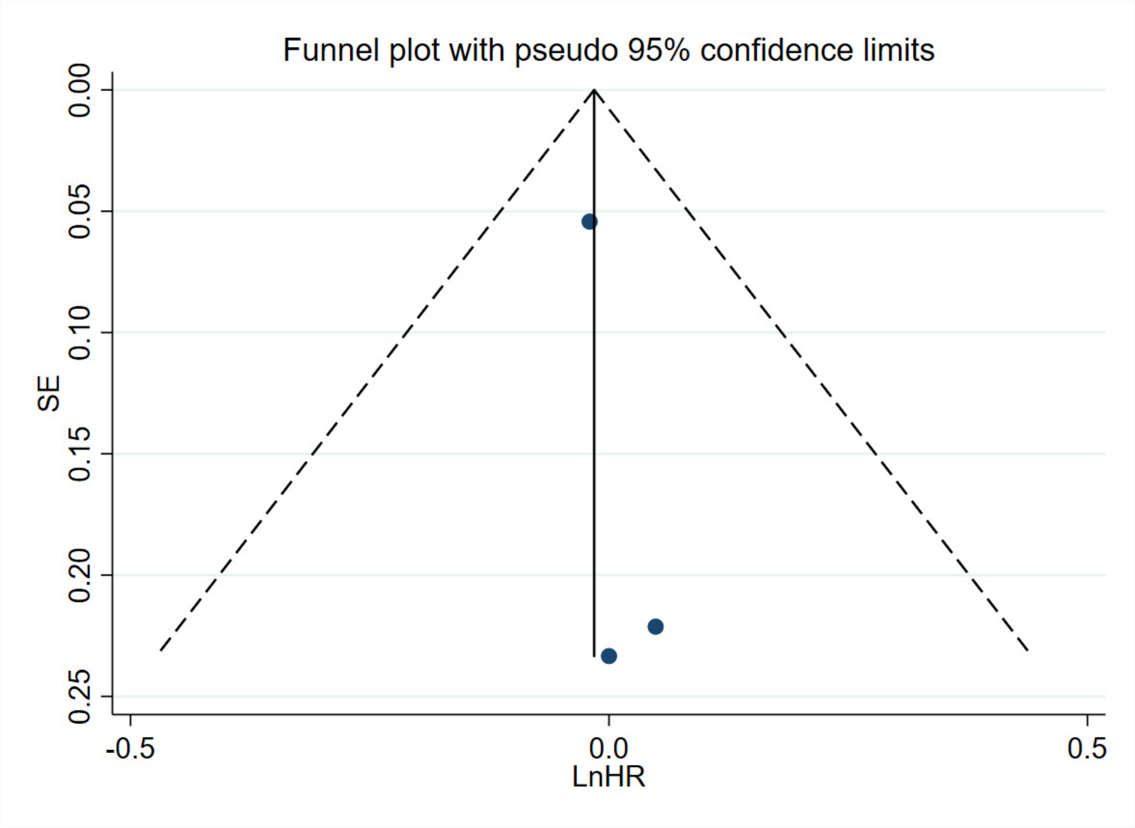

E. Liver cancer.

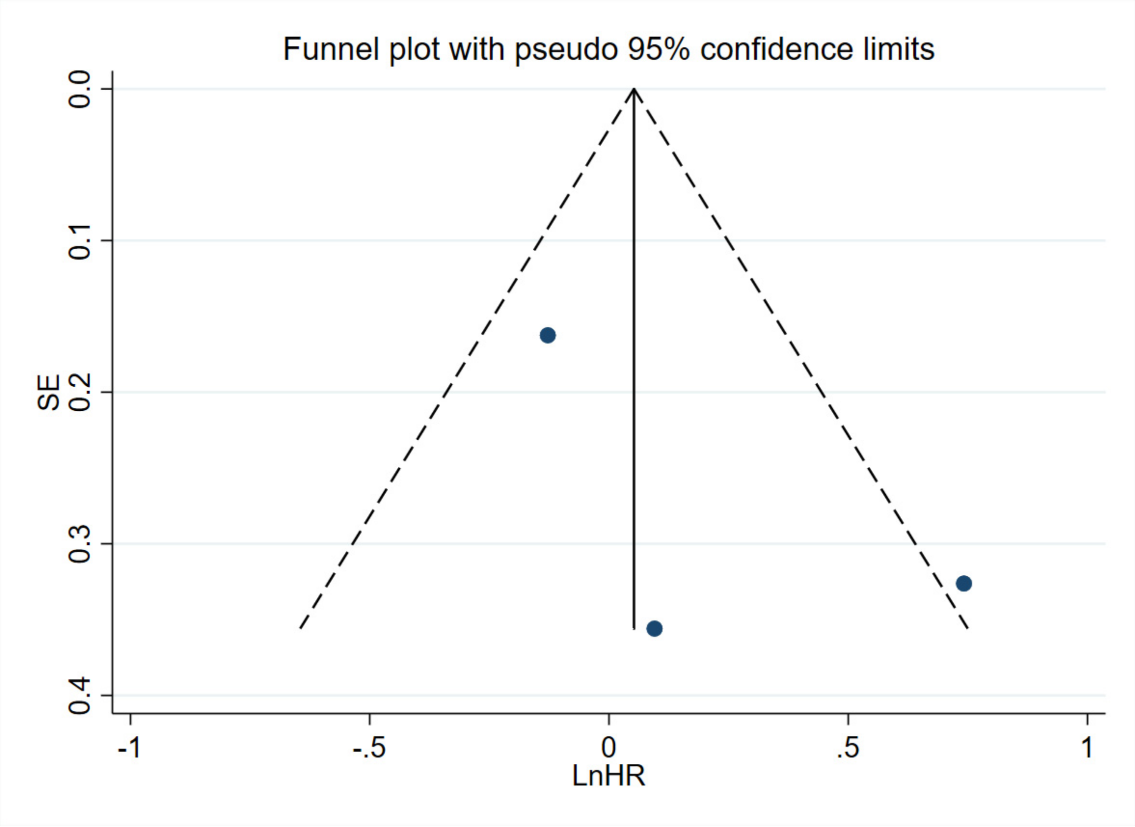

F. Lung cancer.

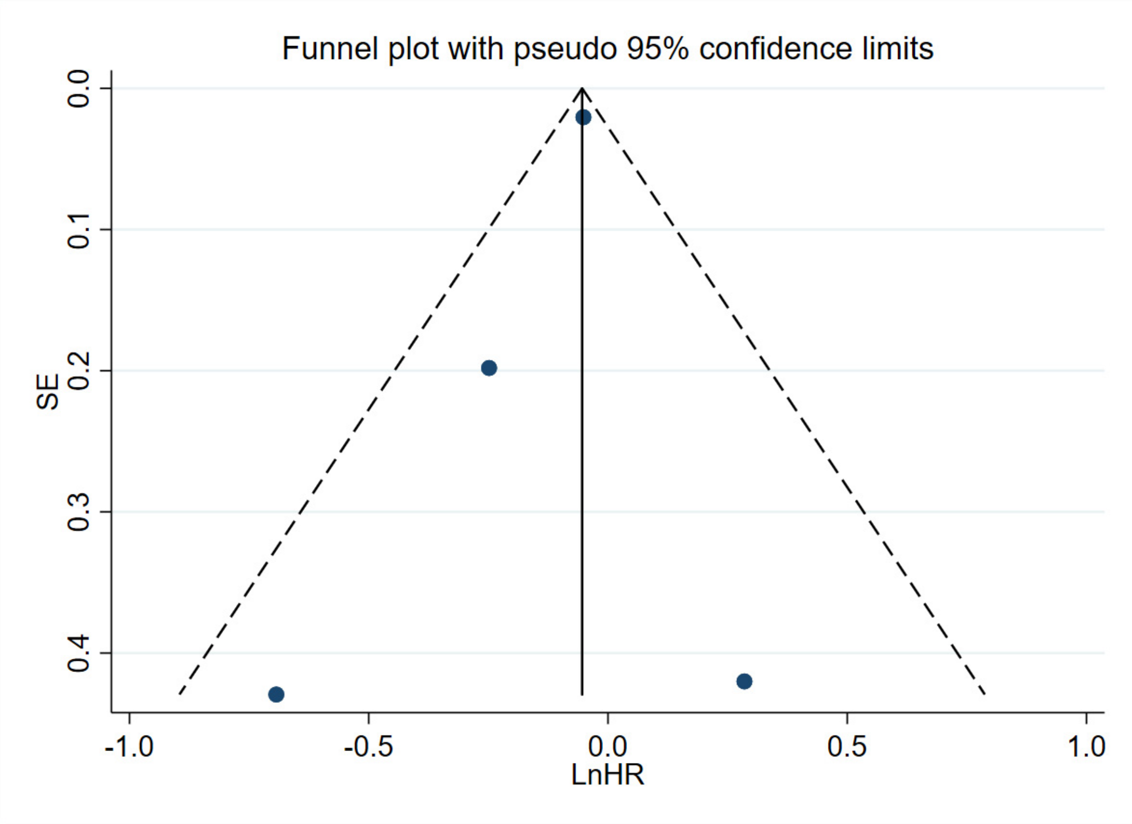

G. Pancreatic cancer.

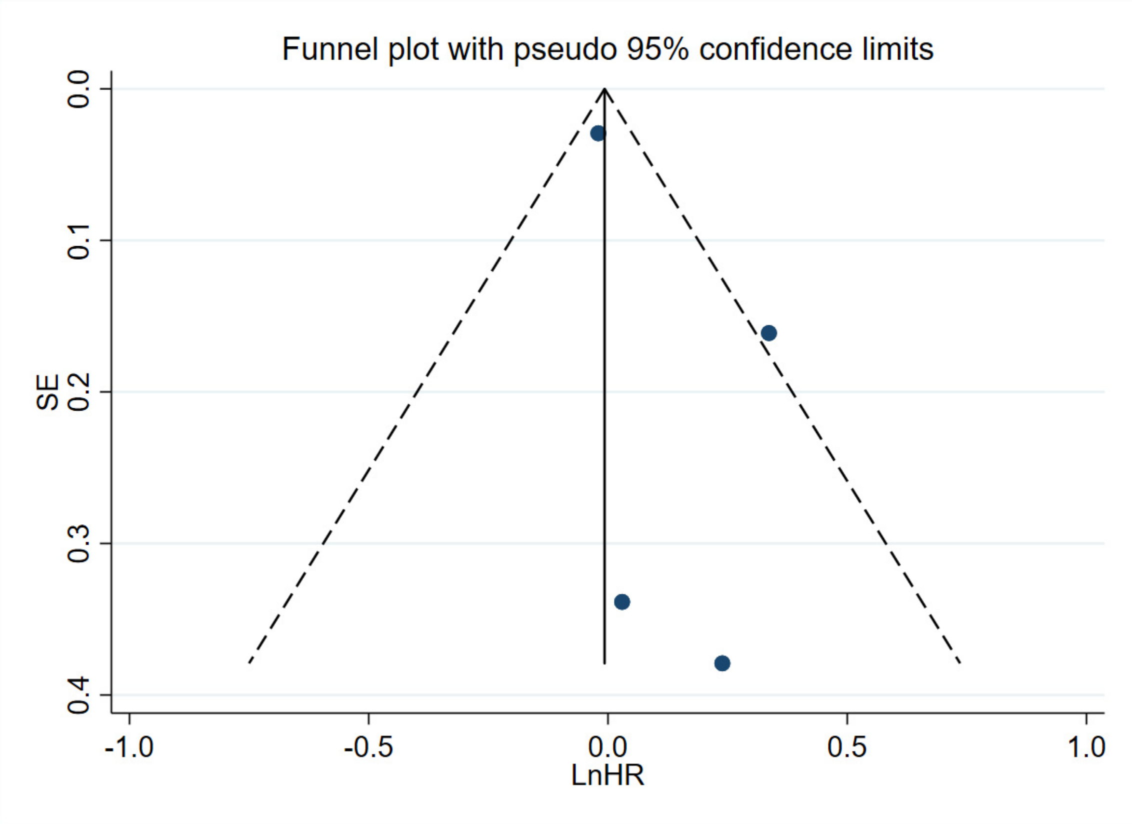

H. Rectum cancer.

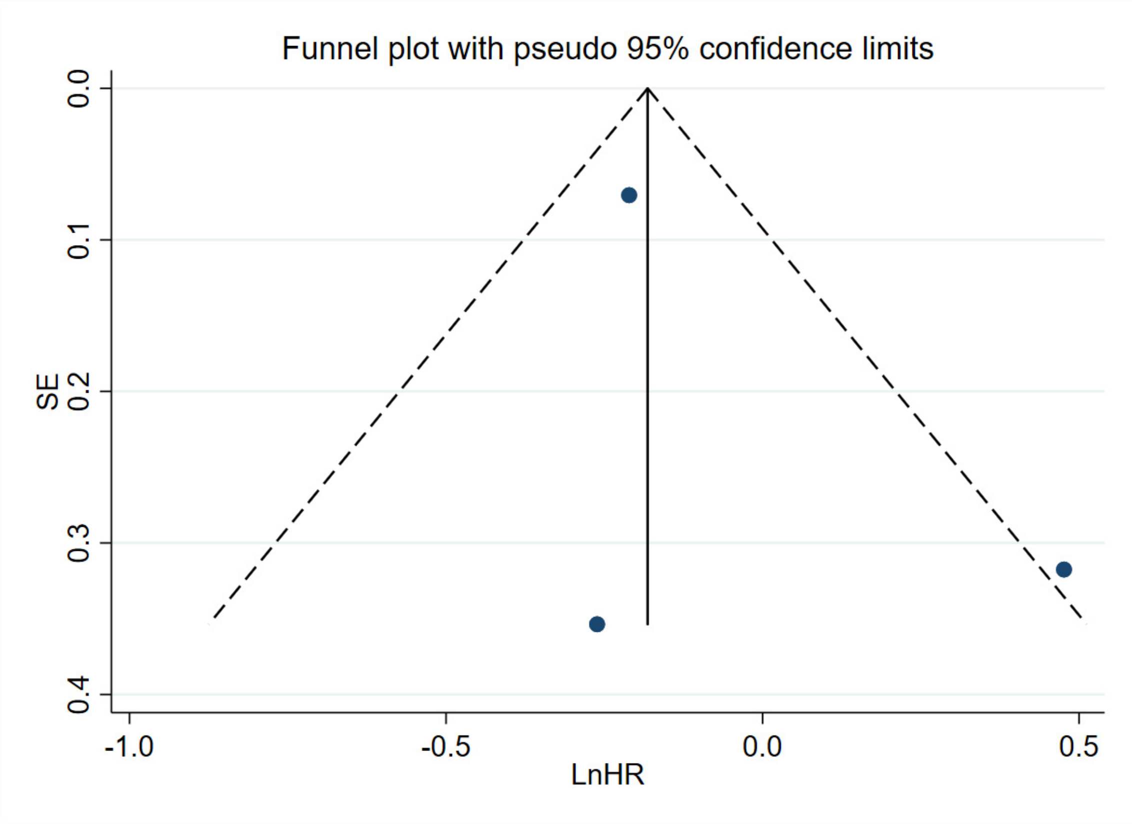

I. Stomach cancer.

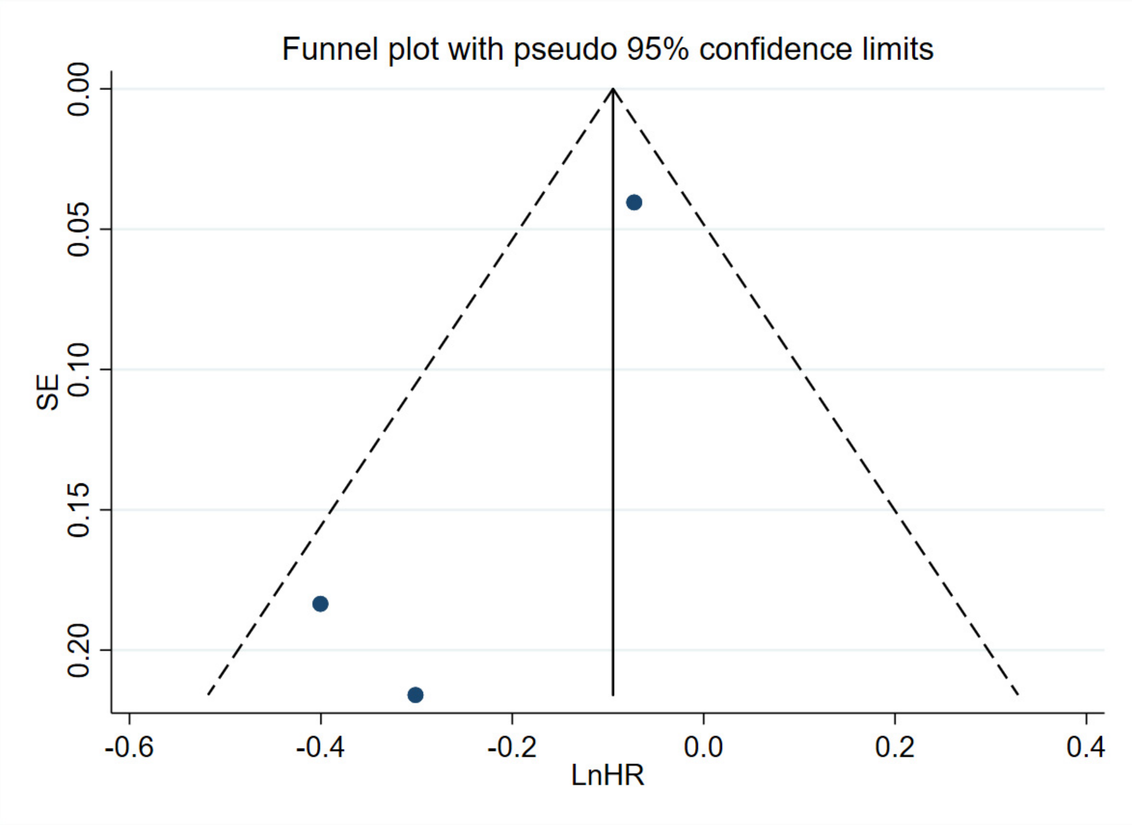

Figure S2. Sensitivity analyses.

A. Overall cancer risk.

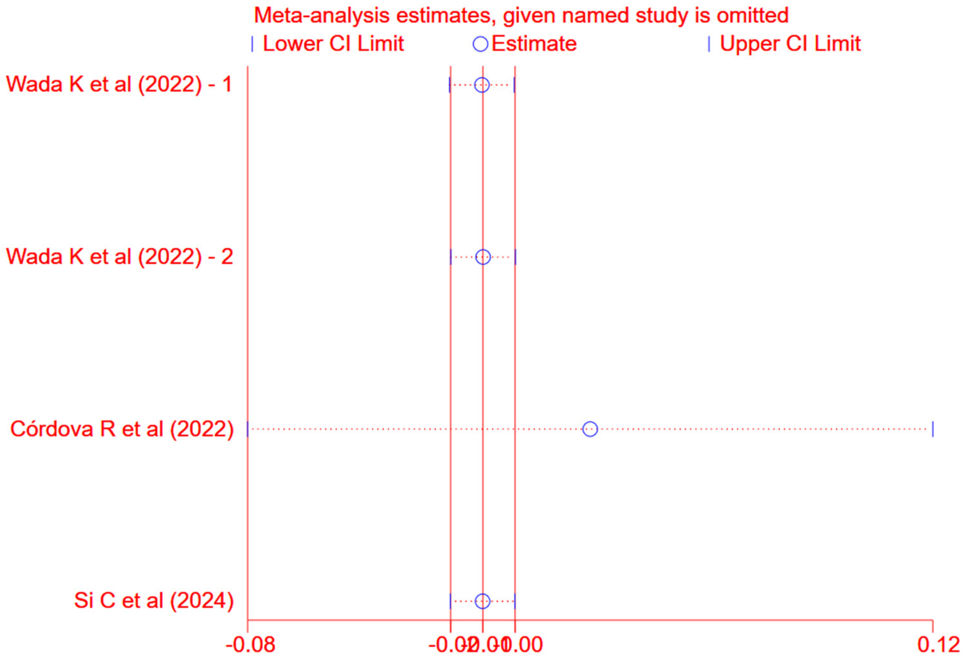

B. Cancer mortality.

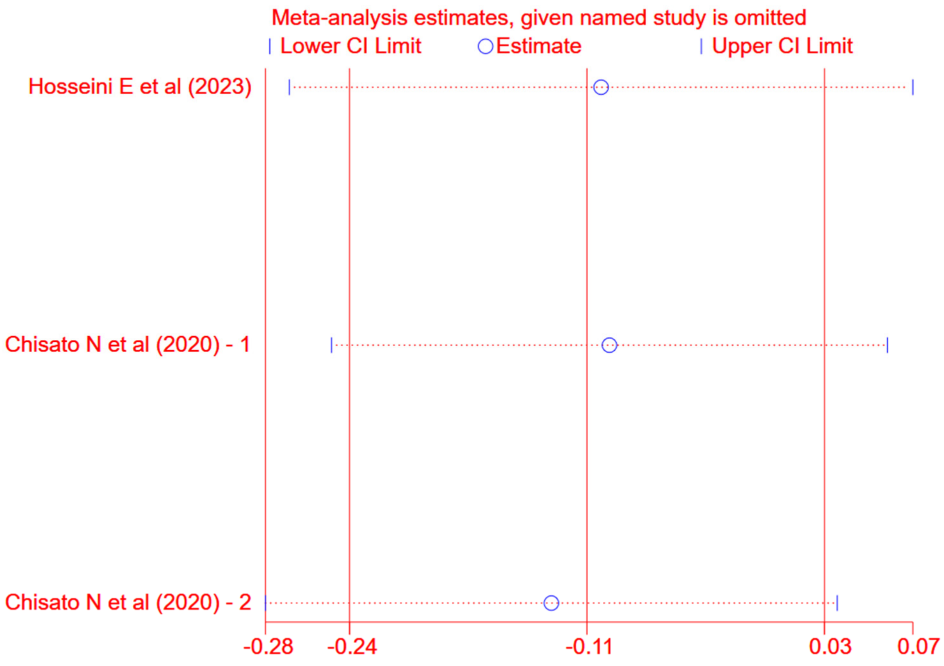

C. Breast cancer risk

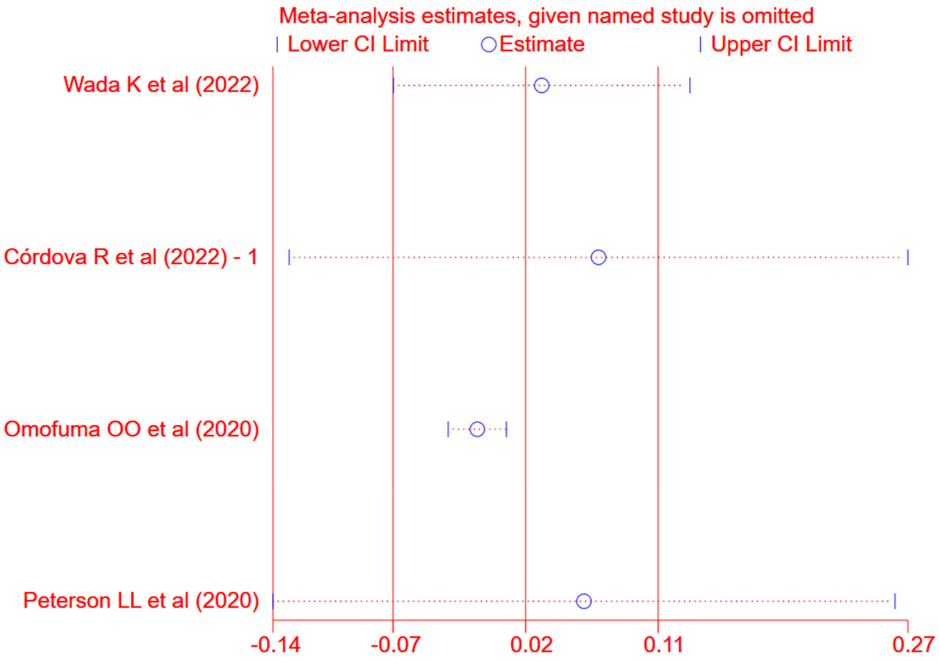

D. Colon cancer.

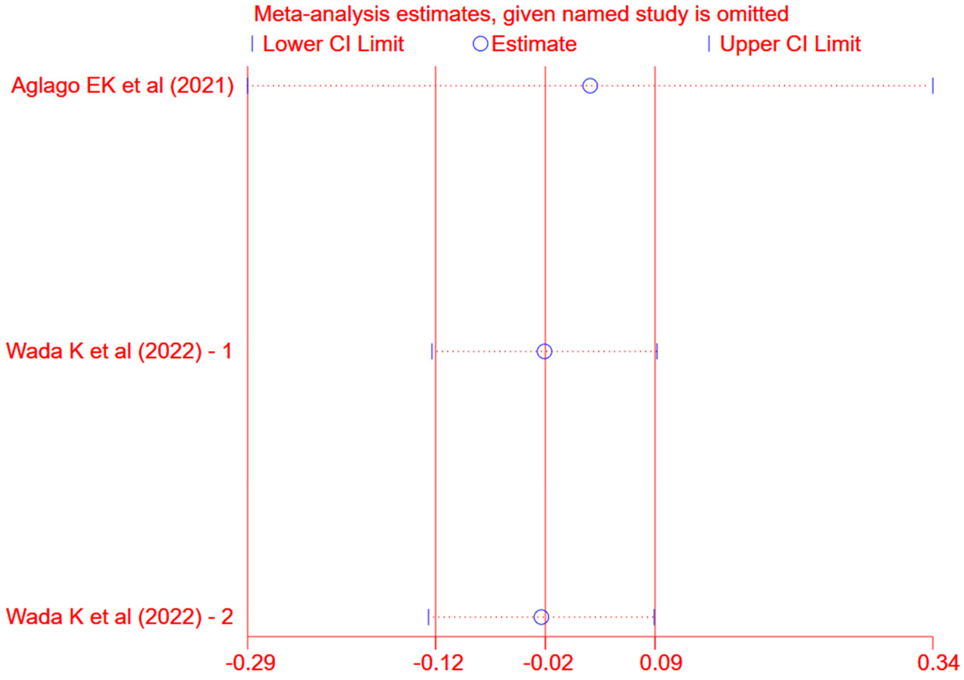

E. Liver cancer.

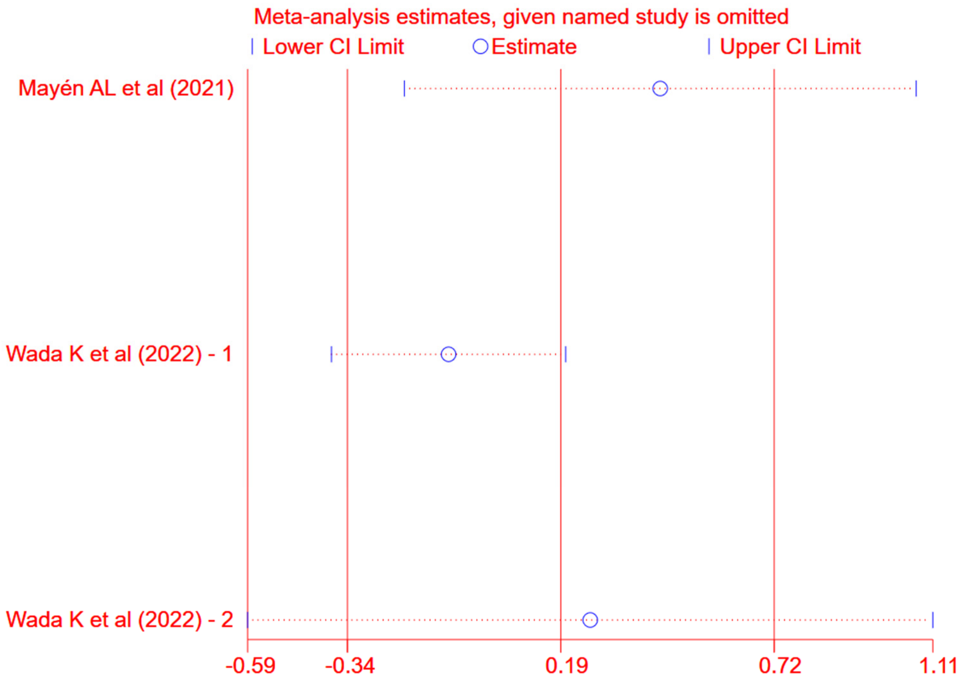

F. Lung cancer.

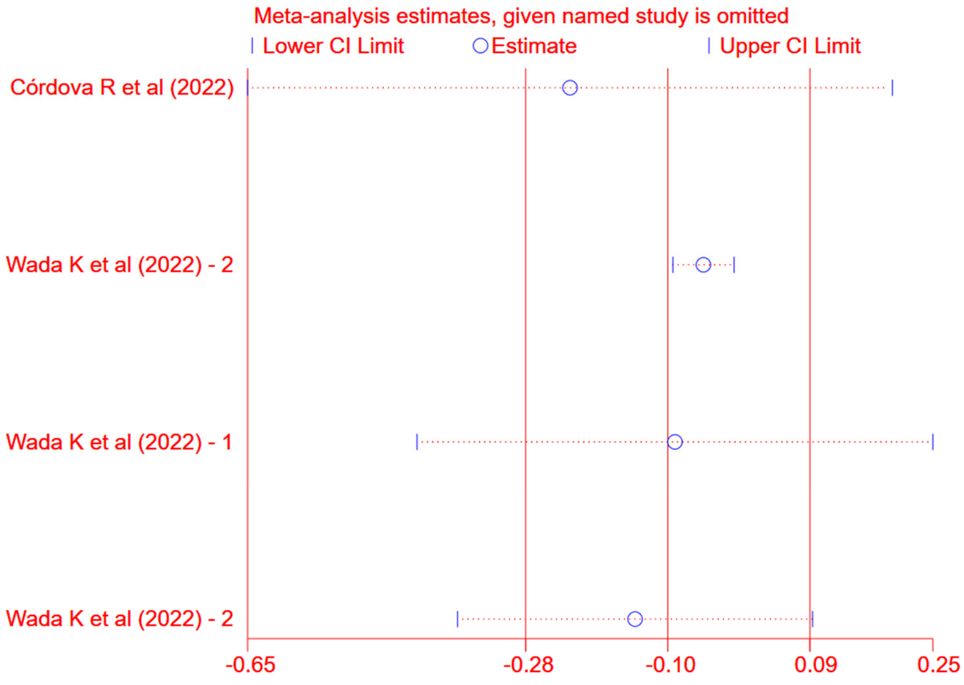

G. Pancreatic cancer.

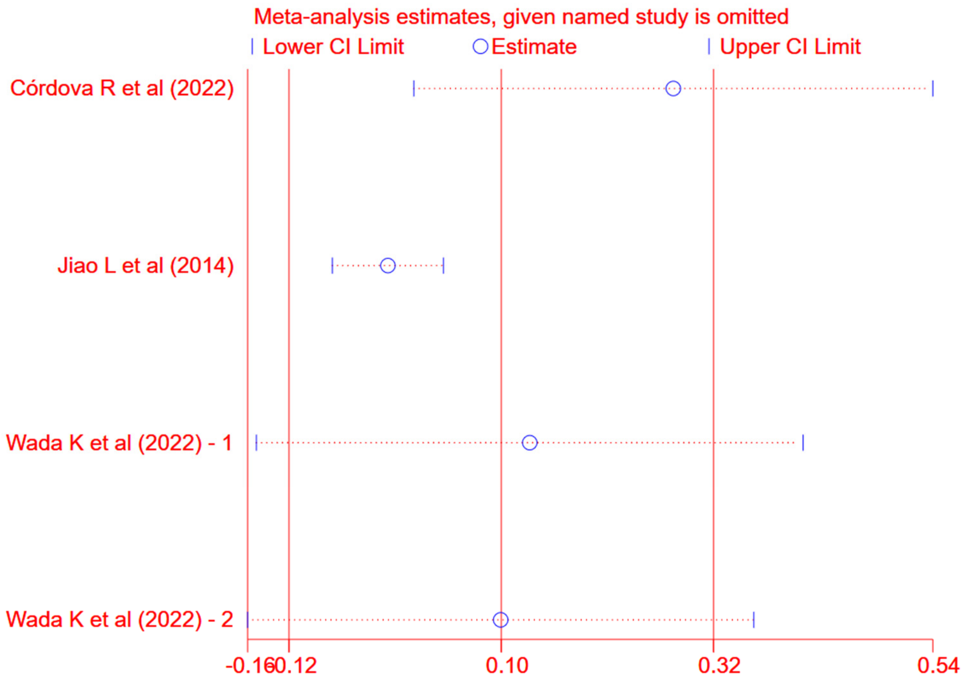

H. Rectum cancer.

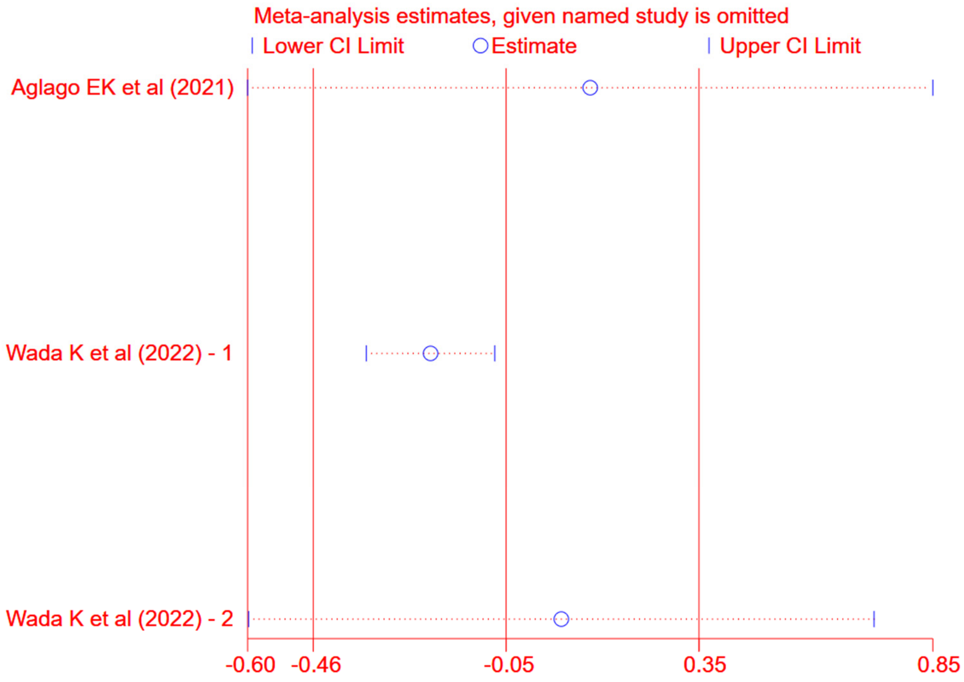

I. Stomach cancer.

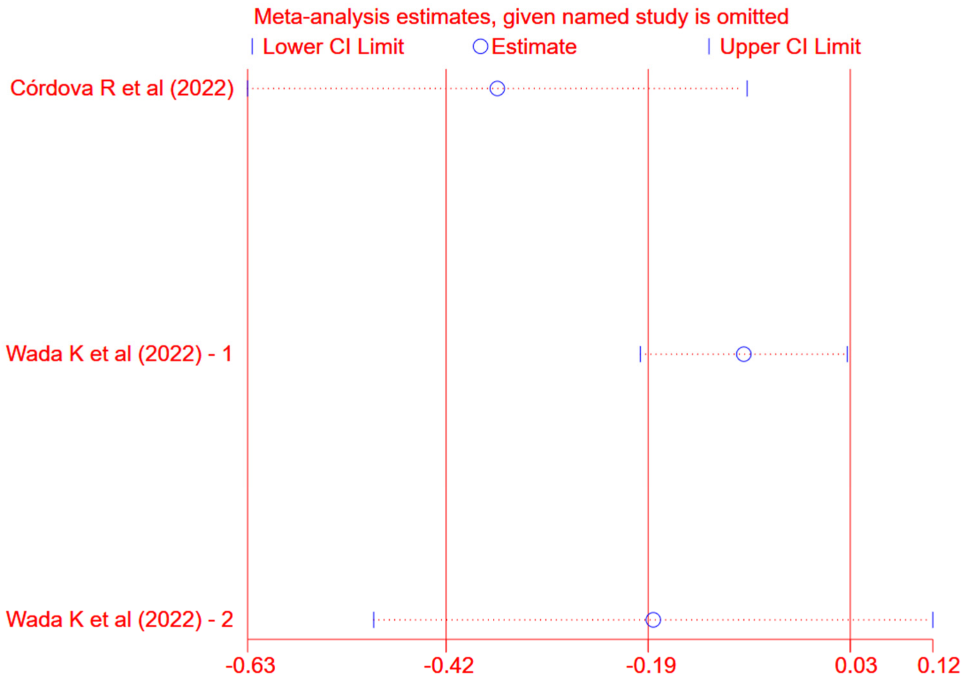

Appendix S1. Search strategy.

- Medline, Scopus, Web of Science, and Cochrane Library

("dietary ages" OR "dietetic ages" OR "dietary advanced glycation end products" OR “advanced glycation end products” OR “dAGE”) AND ("cancer" OR “breast cancer” OR "mortality") AND (risk OR hr OR “hazard ratio” OR “odds ratio” OR “risk ratio”)

- Grey literature

Not specified.
